# Exploring Climate Emotions in Canada’s Provincial North

**DOI:** 10.3389/fpsyg.2022.920313

**Published:** 2022-06-24

**Authors:** Lindsay P. Galway, Thomas Beery

**Affiliations:** ^1^Department of Health Sciences, Lakehead University, Thunder Bay, ON, Canada; ^2^Faculty for Teacher Training, Man and Biosphere Health Research Group, Kristianstad University, Kristianstad, Sweden

**Keywords:** climate change, emotion, climate emotions, climate emotion scale (CES), climate anxiety, Northern, Provincial North

## Abstract

The mental and emotional dimensions of climate change are increasingly concerning as extreme events become more frequent and severe, ecosystem destruction advances, and people become more aware of climate impacts and injustices. Research on climate emotions has rapidly advanced over the last decade with growing evidence illustrating that climate emotions can impact health, shape climate action, and ought to be considered in climate change communication, education, and engagement. This paper explores, describes, and discusses climate emotions in the context of Canada’s Provincial North: a vast region characterized by a vulnerability to climate change, remoteness, political marginalization, diverse Indigenous populations, and economies/livelihoods tied to resource extraction. Using postal survey data collected in two Provincial North communities (Thunder Bay, Ontario, and Prince George, British Columbia; *N* = 627), we aim to (1) describe climate emotions experienced in the context of Canada’s Provincial North, including relationships among specific emotions; and (2) examine if socio-demographic variables (gender, age, and parenthood) show a relationship with climate emotions. Results show high levels of emotional response to climate change overall, with worry and frustration as those emotions reported by the highest percentage of participants. We also find significant difference in climate emotions between men and women. A methodological result was noted in the usefulness of the Climate Emotion Scale (CES), which showed high reliability and high inter-item correlation. A notable limitation of our data is its’ underrepresentation of Indigenous peoples. The findings contribute to a greater understanding of climate emotions with relevance to similar settings characterized by marginalization, vulnerability to climate change, urban islands within vast rural and remote landscapes, and economies and social identities tied to resource extraction. We discuss our findings in relation to the literature and outline future research directions and implications.

## Introduction

The mental and emotional dimensions of climate change are increasingly relevant as extreme events become more frequent and severe, ecosystem destruction advances, and people become increasingly aware of climate impacts and injustices (Clayton et al., 2017; Hayes et al., 2018; Ojala et al., 2021). Over the last decade, interest and engagement in the emotional impacts of, and responses to, climate change has grown substantially (Pihkala, 2022a). This growing interest is evident in scholarly spaces, the mainstream media, and society at large. For example, in 2021, climate anxiety was shortlisted for the Collins Dictionary word of the year (Collins Dictionary, 2021), while influential media outfits across the globe have published stories on topics such as climate grief (Pihkala, 2022b) and climate emotions (Henriques, 2019), and solastalgia (Michelin, 2020). The growing interest in climate emotions is fueled mainly by the urgency of the climate crisis and the closing window of opportunity for preventing the worst of climate impacts and injustices (Masson-Delmotte et al., 2021; Pörtner et al., 2022).

The emerging climate emotions literature reflects the long-overdue recognition that emotions are related to climate change in several important ways (Ray, 2020). Specifically, there is growing evidence illustrating that climate emotions can impact mental health and well-being, are connected to climate action and resilience, and should be considered in climate change communication, education, and engagement efforts (Wang et al., 2018; Bouman et al., 2020; Ray, 2020; Galway et al., 2021; Pihkala, 2022a). The emerging literature is also illustrative of an “affective turn” (Clough and Halley, 2007) concerning climate change. Before describing the climate emotions scholarship further, it is crucial to explicitly articulate what is meant by the term “climate emotions” herein. Following Pihkala (2022a), climate emotions are understood broadly as feelings, emotions, and affects (i.e., affective phenomena) related to, and shaped by, climate change and climate injustices. Climate emotions research is relatively new, rapidly evolving, methodologically and theoretically diverse, and applied (i.e., a strong orientation toward action and policy). Scholars engaging in climate emotions research represent diverse disciplinary perspectives including, but not limited to, public health (Galway et al., 2019; Bratu et al., 2022), communication (Bloodhart et al., 2019), psychology (Clayton and Karazsia, 2020; Gibson et al., 2020; Coffey et al., 2021), sustainability studies (Verlie, 2019), and education (Ojala, 2015; Russell and Oakley, 2016) illustrating the highly interdisciplinary nature of climate emotions research.

To date, five key streams of empirical inquiry characterize the climate emotions scholarship:

1. Describing and understanding experiences of climate emotions.
2. Assessing the impacts of climate emotions on health and well-being.
3. Examining if and how emotions influence climate action, engagement, and/or policy-support.
4. Examining the influences of emotions on risk perception and climate communication.
5. Exploring how emotions relate to climate change education.

Specific studies often advance more than one of these key streams of inquiry. The study presented here falls within and contributes to the first stream of inquiry with a focus on describing and understanding experiences of climate emotions in a specific place, Canada’s Provincial North, such that the literature related to describing and understanding experiences of climate emotions is described and discussed more fully in the following paragraphs.

Because climate emotions are relatively novel affective phenomena, empirical research describing and understanding experiences of climate emotions is essential to ground analytical, theoretical, and practical advances in peoples lived and embodied experiences. The research focused on describing and understanding experiences of climate emotions is generally descriptive and often employs qualitative data collection such as interviews (Wright and Nyberg, 2012; Kemkes and Akerman, 2019; Soutar and Wand, 2022). For example, Kemkes and Akerman (2019) used semi-structured interviews and interpretive phenomenological analysis to examine the lived experiences of climate change on the South shore of Lake Superior, focusing on feelings and emotional responses to climate change. The authors describe how feelings of helplessness and fear emerge from a growing awareness of the urgency, scale, and complexity of climate change while also arguing that processing these difficult emotions can lead to feelings of hope. In another example of a study employing interviews, Jones and Davison (2021) interviewed 21 young adults in Tasmania to analyze emotions associated with climate change. Using thematic analysis, a diversity of climate emotions were identified, including frustration, helplessness, anxiety, and hopefulness (about a better future), illustrating that “participant knowledge of climate change was bound up with an intricate web of emotions” (Jones and Davison, 2021). Specific populations that have received particular attention in qualitative research exploring the lived experiences of climate emotions include youth/young adults (Hiser and Lynch, 2021; Jones and Davison, 2021), those living close to the land (Cunsolo Willox et al., 2013; Ellis and Albrecht, 2017; Howard et al., 2020), and climate activists/scholars (Martiskainen et al., 2020; Halstead et al., 2021).

Surveys have also been used to describe and advance understanding of climate emotions within specific populations, most commonly implemented at the national level (Leviston et al., 2014; Smith and Leiserowitz, 2014; Minor et al., 2019; SITRA, 2019; Leiserowitz et al., 2021a). Studies employing survey methods often also include an analytical dimension examining associations between climate emotions and endpoints such as climate action (Bouman et al., 2020), communication (Brosch, 2021), or health (Stanley et al., 2021). Specific climate emotions that, to date, are most commonly reported in existing literature include worry, frustration, anger, sadness, helplessness, and hopefulness (Clayton et al., 2017; Hickman et al., 2021). According to representative survey data from the United States collected in 2021, approximately 70% of Americans report that they are “somewhat” or “very” worried about global warming (Leiserowitz et al., 2021a). Approximately four in ten Americans report feeling angry (47%) or hopeful (42%). A 2019 national survey of the emotions evoked by climate change in Finland (2019) found that feelings of frustration, powerlessness, anger, fear, and grief were most commonly reported (SITRA, 2019). A large multinational study that surveyed 10,000 young adults (16–25 years) in 10 nations aimed to “better understand the feelings, thoughts, and functional impacts associated with climate change among young people globally” (Hickman et al., 2021). In this study, feeling worried, sad, afraid, and anxious were reported by at least 60% of respondents, while feeling angry, powerless, and helpless were reported by at least 50% of respondents. These findings illustrate that myriad climate emotions are prevalent among general populations at national levels and likely heightened among young people.

In this existing literature, specific climate emotions are often positioned and described as either “positive” or “negative” (Wang et al., 2018; Bloodhart et al., 2019; Schneider et al., 2021). From this perspective, “negative” climate emotions include worry, anger, fear, powerlessness, and frustration, while hope and resilience are most commonly positioned and discussed as “positive” emotions (Schneider et al., 2021). There is also emerging work developing tools and multi-item scales for measuring climate emotions, which will play an important role in the research field. Since the survey collection for this study, Clayton and Karazsia (2020) and Wullenkord et al. (2021) have published validated scales in English and German focused on climate anxiety, for example.

Given that climate emotions research is relatively new and rapidly emerging, many research gaps, unanswered questions, and debates remain (Pihkala, 2022a). Notable gaps in the literature focused on describing and understanding climate emotions include the need for research across a greater diversity of people and places, research beyond the United States, Australia, and Europe in particular. Like climate change broadly, climate emotions are experienced “very differently by different people” (Gobby, 2020); more research is needed to understand better “when, where, for whom, and under which conditions” climate emotions are experienced (Galway et al., 2019). Exploring climate emotions in specific places is also necessary, given that climate change is a global phenomenon with local and regional impacts and is understood and experienced in places (Hess et al., 2008; Cunsolo Willox et al., 2012; Adger, 2016; Döring and Ratter, 2017; Beery, 2018; Galway, 2019).

There is also a blind spot in survey-based research on climate emotions outside of urban settings and at local and regional levels (Soutar and Wand, 2022). Moreover, more work exploring the interconnectedness among specific emotions is needed to inform future developments related to frameworks and taxonomies/typologies of climate emotions. As noted above, developing reliable scales to measure and monitor climate emotions is also needed. Finally, research around emotions and affect generally has shown that socio-demographic variables such as gender, age, and income are influential factors when aiming to describe and understand emotional experiences generally, emphasizing the need for research aimed at understanding how socio-demographic factors are related to climate emotions specifically (Bloodhart et al., 2019). In other words, many questions remain about who experiences which kinds of climate emotions.

The present study is informed and inspired by the emerging climate emotions research as well as the trends and the critical knowledge gaps and unanswered questions described above. Specific aims of this study are to (1) describe climate emotions experienced in the context of Canada’s Provincial North, including relationships among specific emotions; and (2) examine if socio-demographic variables (gender, age, and parenthood) show a relationship with climate emotions.

## Materials and Methods

### Study Context

The study reported herein was one dimension of a multi-year project focused on communicating climate change impacts and solutions in ways that promote engagement with climate action in the context of Canada’s Provincial North. The larger project used a place-based approach and employed mixed-methods in the context of two regional case-study settings of the Provincial North: Northern BC and Northern Ontario. Canada’s Provincial North is a regional band south of the 60th parallel and north of the Canada-United States border that extends across the country from Labrador on the East to British Columbia on the West. The region is characterized by remoteness, political marginalization, underemployment, diverse Indigenous populations, resource–dependence, and heightened vulnerability to climate change (Southcott and Irlbacher-Fo, 2009; Wiersma and Koster, 2013; Galway, 2019; Gislason et al., 2021). Indigenous and settler peoples residing in this region also exhibit resistance and resilience to ongoing disturbances and boom and bust economies alongside strong connections with the land and nature. We aim to address the complete lack of climate emotions research in this setting. Coates and Morrison (1992) and Coates et al. (2015) describe Canada’s Provincial North as the “Forgotten North,” this description is certainly fitting with regards to climate emotions research specifically and climate change research generally. The findings will be particularly relevant to communities across the provincial North of Canada and relevant to similar settings characterized by remoteness, heightened vulnerability to climate change, dependence on resource extraction, and a strong sense of connectedness to land and nature.

### Research Design

The study employed an exploratory quantitative design to investigate the noted gaps in climate emotions research. The quantitative dimension of the project used postal surveys to document public beliefs, attitudes, and perceptions of climate change and climate action. The postal surveys were implemented in two communities within the regional case-study setting: Thunder Bay (Northern Ontario) and Prince George (Northern British Columbia). See Galway et al. (2021) for a more complete description of these two communities. This paper uses the data collected via these postal surveys to explore and enhance understanding of climate emotions in the context of Canada’s Provincial North. The design allowed for the use of data to describe climate emotions experienced in the context of Canada’s Provincial North. Moreover, the design facilitated correlational analysis, including relationships among specific climate emotions and consideration of whether socio-demographic variables show a relationship with climate emotions.

### Data Collection and Sample

Development of the survey instrument was informed by surveys previously implemented by the Yale Program on Climate Change Communication (The Yale Program on Climate Change Communication) in collaboration with members of a Research Advisory Group (i.e., community members and key informants in each study community). The survey instrument was pilot tested with 19 community members to ensure clarity of questions and identify any issues prior to administration. The final instrument consisted of 36 questions using a combination of Likert scale, ranking, fixed-choice answers, and open-ended questions in five main categories: (i) perspectives on climate change in general; (ii) climate change impacts and emotions; (iii) climate action; (iv) connectedness to nature; (v) socio-demographic questions. The instrument is available from authors on request. The Research Ethics Boards approved this research at Lakehead University, the University of Northern British Columbia, and Simon Fraser University prior to data collection.

The postal survey was distributed by Canada Post mail to 4,000 randomly selected households thereby gathering data from a representative cross-section of adults (over the age of 18). A simple random selection of households was selected from all addresses using the Canada Post address database. Canada Post randomly selected 2,000 households in Prince George and 2,000 households in Thunder using the Census Metropolitan Areas as sampling frames (populations of 86,622 and 121,621 for respectively). To increase response rates, the Dillman Tailored Design Method (Dillman et al., 2014) was adapted. The survey was administered in early 2019 and involved three waves of mailing. First, a survey packets containing the survey instrument, an information letter, and a pre-paid envelope to return the completed survey were sent out. Two weeks later, a reminder postcard was sent. A second and final reminder postcard was sent out another week later, along with information about how to complete the survey electronically.

The information letter explained the survey and encouraged participation by an adult member of the household, age 18 or older. If there was more than one adult in the household, instructions indicated that the person who has had the most recent birthday should complete the enclosed survey. The letter also included information about a random draw ($100 gift card) for those who completed the survey to enhance the response rate. The reminder postcards included information about getting another survey packet if it was never received or was lost.

Of the 4,000 mailed surveys originally mailed out, 192 surveys did not reach selected households and were returned to sender by Canada Post. A total of 693 surveys were completed (76 were completed electronically).

### Data Analysis

Statistical data analysis was conducted using Statistical Package for the Social Sciences, SPSS 27. The first step in the analysis process was to calculate the response rate considering responses, incomplete, and duplicate entries. Descriptive statistics were then calculated using the complete data set analysis. Eleven emotions were measured with a single Likert-scale item, “How strongly do you feel each of the following emotions when you think about climate change?” ranging from *Very strongly* (4) to *Not at all* (1). The specific emotions are listed in Table 1. Initial analysis also included a Cronbach’s Alpha for the eleven items making up the climate emotion scale; as follow-up to the Cronbach’s alpha and to further consider the strength of the CES, a confirmatory factor analysis was conducted. Further, a series of *t*-tests were run to compare the CES mean scores between men and women and between participants with children (or intending to have children) and those without children. A correlation coefficient was computed between age/generation categories and the CES to test for a bivariate correlation.

**TABLE 1:** Pearson’s correlation matrix for all emotion variables^1^.

	Worry	Frustration	Fear	Anger	Sadness	Helpless	Hopeless	Guilt	Anxiety	Hope
Frustration	0.654**									
Fear	0.708**	0.618**								
Anger	0.594**	0.750**	0.681**							
Sadness	0.670**	0.627**	0.676**	0.680**						
Helpless	0.486**	0.549**	0.555**	0.537**	0.587**					
Hopeless	0.483**	0.473**	0.560**	0.538**	0.567**	0.754**				
Guilt	0.492**	0.487**	572**	0.523**	0.548**	0.481**	0.545**			
Anxiety	0.548**	0.534**	0.657**	0.592**	0.576**	0.466**	0.532**	0.614**		
Hope	0.164**	0.145**	0.206**	0.146**	0.181**	0.065	−0.003	0.142**	0.189**	
Resilience	0.047	0.053	0.081	0.059	0.087	−0.013	−0.050	0.060	0.153**	0.569**
*M*	3.09	2.90	2.56	2.60	2.80	2.81	2.51	2.20	2.28	2.40
*SD*	0.811	0.965	0.932	0.982	0.977	0.963	0.939	0.902	0.936	0.860
*N*	623	615	624	620	623	619	622	623	619	618

^1^Correlations are reported for all participants answering yes to the question: Do you think that climate change is happening? **Correlation is significant at the <0.001 level (two-tailed).

## Results

### Survey Results

Two trained research assistants entered data from the postal surveys into a Qualtrics electronic database to reduce the likelihood of data entry errors. A first step in the analysis process was an initial review of the data for missingness; 38 survey responses were excluded as a result of being incomplete (more than 50% of responses left blank) or duplicate entries resulting in a complete data set of 655 surveys, an adjusted response rate of 17.2%. Additionally, the final dataset was reviewed after data entry for any errors. For the present study, we excluded any respondents that did not answer “yes” to the following screening question: “*do you think that climate change is happening*.” Therefore, the final sample used in this study was *N* = 627. The average participant age of these 627 participants was 57 years, with an average community residency of 40 years. Three-hundred fifty-six respondents were female (57%), and 258 were male (41%). Compared to 2016 Census data, our sample slightly over-represents older women. Ninety percent of respondents were white, and no other racial or cultural grouping was greater than 2% such that our sample underrepresents individuals who self-identify as Indigenous compared to 2016 Census data. Four hundred-ninety participants (79%) indicated that they had or intended to have children; 127 participants did not have children (20%).

**FIGURE 1:**
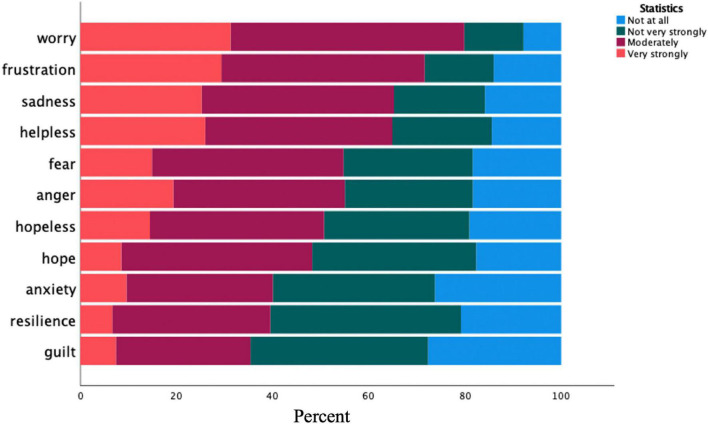
Reporting levels of specific climate emotions (in percent) (*N* = 655).

Eleven climate emotion variables were used to explore participants’ emotional responses to climate change (worry, frustration, sadness, helplessness, fear, anger, hopelessness, hope, anxiety, resilience, and guilt) and the noted CES. Participants were asked to indicate how strongly they experienced the series of emotions when thinking about climate change (rated along a 4-point scale from 1 = Not at all, to 4 = Very strongly). At least 50% of survey respondents indicated a moderate and strong emotional response regarding 7 of the eleven items (worry, frustration, sadness, helplessness, fear, anger, and hopelessness). Using the “moderately” and “very strongly” responses for the survey items, the two most commonly reported emotional responses to climate change were “worry” (82%) and “frustration” (71%). The two items with the lowest reports of moderately or strongly responses were “resilience” (40%) and “guilt” (37%). Descriptive results of the 11 climate emotion items are displayed in Figure 1 and Table 1.

A Cronbach’s Alpha for the eleven items was calculated, and the result, α = 0.89, indicated strong internal consistency (Vaske, 2008). This reliability is supported by the correlation scores for the eleven CES items, as presented in Table 1. Correlation coefficients were calculated among the emotion variables. Using the Bonferroni approach to control for Type 1 error across the 55 correlations, a *p*-value of less than 0.0009 (0.05/55) was required for significance. The results of the correlational analysis presented in Table 1 show that 45 of them were statistically significant at the *r* > 0.001 level and were greater than or equal to 0.14; ten correlations were not significant. Thirty-one of the 45 significant correlations showed a substantial relationship (</= 0.50). The strongest of these correlations included fear and worry *r*(621) = 0.71, *p* < 0.001, anger and frustration *r*(610) = 0.75, *p* < 0.001, and hopeless and helpless, *r*(615) = 0.75, *p* < 0.001.

Based on the results of the Cronbach’s Alpha, an attempt to validate the scale using confirmatory factor analysis was conducted. Initially, the factorability of the 11 climate emotion items was examined. Firstly, all of the 11 items correlated at least 0.3 with at least one other item, suggesting reasonable factorability. Secondly, the Kaiser-Meyer-Olkin measure of sampling adequacy was 0.90, above the recommended value of 0.6, and Bartlett’s test of sphericity was significant [χ^2^(55) = 3855.93, *p* < 0.001]. Given these overall indicators, confirmatory factor analysis was conducted with all 11 items. The initial eigenvalues showed that the first factor explained 53% of the variance, and the second factor 14% of the variance using oblimin rotations of the factor loading matrix. The two-factor solution, explained 67% of the variance, was preferred because of its previous theoretical support, and the “leveling off” of eigenvalues on the scree plot after two factors. All items had primary loadings over 0.7 (see Table 2).

**TABLE 2:** Factor loadings and communalities based on a confirmatory factor analysis with oblimin rotation for 11 items of the Climate Emotions Scale (CES).

	Negative emotions	Positive emotions
Fear	0.84	
Sadness	0.83	
Anger	0.82	
Frustration	0.80	
Hopeless	0.79	
Helpless	0.78	
Worry	0.78	
Anxiety	0.76	
Guilt	0.73	
Resilience		0.88
Hope		0.86

Further analysis of climate emotions across levels of socio-demographic variables of gender, generations, and parental status was conducted; note, we use the term gender vs. sex as participants were asked to report whether they *identified* as men/women/other. An independent samples *t*-test was run to compare the mean scores between men and women on the CES. The 369 female participants (*M* = 2.68, *SD* = 0.68) compared to the 258 male participants (*M* = 2.44, *SD* = 0.68) had a significantly higher score on the CES, *t*(625) = −4.693, <0.001. Table 3 provides a variable-by-variable comparison for the eleven CES scale items across gender.

**TABLE 3:** An independent samples *t*-test results comparing the CES mean scores for men and women.

CES variable	Scale	Male percent	Female percent
Worry	Not at all	71.4	28.6
	Not very strongly	60.5	39.5
	Moderately	39.9	60.1
	Very strongly	31.2	68.8
Frustration	Not at all	59.2	40.8
	Not very strongly	41.1	58.9
	Moderately	39.8	60.2
	Very strongly	35.9	64.1
Sadness	Not at all	57.1	42.9
	Not very strongly	53.3	46.7
	Moderately	34.0	66.0
	Very strongly	34.8	65.2
Helpless	Not at all	57.9	42.1
	Not very strongly	36.6	63.4
	Moderately	41.8	58.2
	Very strongly	36.6	63.4
Fear	Not at all	66.7	33.3
	Not very strongly	46.2	53.8
	Moderately	32.7	67.3
	Very strongly	27.1	72.9
Anger	Not at all	58.8	41.2
	Not very strongly	38.8	61.2
	Moderately	39.4	60.6
	Very strongly	33.3	66.7
Hopeless	Not at all	56.7	43.3
	Not very strongly	30.9	69.1
	Moderately	40.5	59.5
	Very strongly	46.2	53.8
Hope	Not at all	52.4	47.6
	Not very strongly	37.0	63.0
	Moderately	38.7	61.3
	Very strongly	48.1	51.9
Anxiety	Not at all	56.5	43.5
	Not very strongly	39.1	60.9
	Moderately	36.2	63.8
	Very strongly	29.0	71.0
Resilience	Not at all	44.2	55.8
	Not very strongly	38.7	61.2
	Moderately	40.9	59.1
	Very strongly	46.3	53.7
Guilt	Not at all	54.8	45.2
	Not very strongly	39.7	60.3
	Moderately	34.1	65.9
	Very strongly	31.3	68.8

The variable birth year was recoded into specific age categories for a generational consideration of age regarding climate emotions [iGen (1997–, Millennials 1981–1996, Generation X 1965–1980, Baby Boomers 1946–1964, Silent 1928–1945, Greatest before 1928]. Generational groupings have been used given previous use in climate change survey research (Leiserowitz et al., 2021a). The results of the bivariate correlation analysis did not show a significant relationship between the variable of age and the CES.

An independent samples t-test was run to compare the mean scores between participants with children (or intending to have children) and those without children. The 490 participants (*M* = 2.6, *SD* = 0.63) with children compared to the 127 participants without children (*M* = 2.6, *SD* = 0.68) displayed no significant difference on the climate emotion response scale *t*(615) = 0.95, *p* = 0.33.

### Limitations

There are several limitations that should be recognized when interpreting findings and considering implication of this research. This study is mainly descriptive and the examined relationships are correlational. Although we examined 11 specific climate emotions, there are likely specific emotions not included in our study that are also being experienced. Other research has included grief, powerlessness, and resentment (Verlie, 2019; Hickman et al., 2021). Further we acknowledge that data collection was conducted at one point in time; emotional responses and impacts associated with climate change are likely transient and dynamic over time (Brosch, 2021). Our sample population somewhat overrepresents older adults (older than 55) and individuals below the age of 18 were not eligible to participate in our study, which may have implications for our findings. Future research with youth in the Provincial North and similar settings is needed given that this sub-group is likely at heightened risk of experiencing mental and emotional health consequences of climate change (Hickman et al., 2021). Additionally, although our data adequately represents the study communities in terms of gender (compared to 2016 Census profiles), our data are not representative of the study communities in terms of race. Specifically, individuals self-identifying as Indigenous are underrepresented in our data. Future research focused on the mental and emotional dimensions of climate change, drawing on Indigenous methodologies (rather than survey-based research) and working *with* Indigenous communities and peoples in the Provincial North, is called for to address this limitation (Kovach, 2021). Finally, although these findings are likely generalizable to people and places across Canada’s Provincial North and communities in similar settings, they may not hold in highly urbanized areas or in more rural and remote communities.

## Discussion and Conclusion

Knowledge and understanding of how people react to and feel about environmental degradation, including climate change, is a growing imperative in the context of the Anthropocene (Albrecht, 2019; Ojala et al., 2021). The feelings, emotions, and affects that are related to, and shaped by, climate change and climate injustices are increasingly experienced and reported across diverse cultures and places worldwide (Albrecht, 2019; Hickman et al., 2021; Ojala et al., 2021; Pihkala, 2022a). There is growing evidence illustrating that climate emotions can impact health, shape climate action, and ought to be considered in climate change communication, education, and engagement (Ray, 2020; Berry and Schnitter, 2022; Pihkala, 2022a). This growing evidence underscores the importance of understanding how people react to and feel about the climate crisis in specific places. Given that empirical research exploring climate emotions is non-existent in the Provincial North of Canada (and also lacking from similar non-urban settings characterized by remoteness, political marginalization, diverse Indigenous populations, strong connectedness to the land/nature, resource–dependence, and heightened vulnerability to climate change), a core aim of this study was to describe climate emotions in this unique context. Moreover, the study examined differences in climate emotions by gender, age, and parental status.

According to our findings, using postal survey data from 627 adults in two communities in Canada’s Provincial North, adults report high levels of climate emotions indicative of emotional distress due to the climate crisis. This distress is likely shaped and fueled by future-oriented concerns, witnessing climate impacts locally and worldwide, low levels of governmental climate action in Canada, and direct experiences of impacts given the increasing prevalence of climate-related extreme events (flooding and wildfires in particular) in the study region. In other words, the high levels of emotional distress identified here are likely influenced by direct and indirect/vicarious pathways (Ojala et al., 2021; Berry and Schnitter, 2022). At least half of the survey respondents indicated moderate or strong feelings of worry, frustration, sadness, helplessness, fear, anger, and hopelessness. These findings emphasize the need for mental and emotional health programs and initiatives to ensure that people across the Provincial North (and beyond) have opportunities to process and discuss climate emotions and the support needed to cope with strong emotions in ways that are healthy and translate into engagement and action. As Ray (2020) argues, collective activities, where people come together in a safe space to engage in supportive processes to recognize and process strong and complex emotional responses to climate change, are called for to build individual and collective resilience.

An early framework for thinking about emotional impacts of environmental risks developed by Böhm (2003) differentiated between consequence-based retrospective emotions (e.g., sadness), consequence-based prospective emotions (e.g., worry and hope), ethics-based self-focused emotions (e.g., guilt) and ethics-based other-focused emotions (e.g., anger). Our findings illustrate that consequence-based retrospective and prospective emotions are commonly experienced in the Provincial North of Canada, while ethics-based emotions are less common. Future research using qualitative data collection such as interviews and focus groups may help elucidate why consequence-based emotions are more common than ethics-based emotions in this study context with implications for developing appropriate and supportive programs and initiatives. Moreover, although few examples of mixed methods research focused on describing and understanding experiences of climate emotions exist to date, this may be a generative approach design for future research.

Worry and frustration were the most reported emotional responses (82 and 71%, respectively, reported at least moderately); these two specific emotions have also been identified as the most commonly reported emotional impacts of climate change according to survey data in the United States (Leiserowitz et al., 2021b) and Finland (SITRA, 2019). Notably, the percentage of Provincial North participants indicating worry about climate change is 12% higher than the general American public (Leiserowitz et al., 2021a). Worry has been a core focus in climate emotions research (Bouman et al., 2020). The importance of worry in this study is also supported by previous work considering the role worry plays in climate action (Galway et al., 2019), where worry was shown to function as a mediator in the relationship between connectedness to nature and individual-level climate action. Worry is of particularly interest in health research on climate emotions (Davey and Wells, 2006; Tully et al., 2013). High levels of climate worry can become pathological, ultimately leading to consequences for overall health and well-being for some people and populations (Ogunbode et al., 2021). Although worry is a particularly important climate emotion in Canada’s Provincial North and beyond, we emphasize that it is also essential to recognize, consider, and process a range of interconnected emotions, as illustrated in the CES.

A high Cronbach’s Alpha was seen for all eleven climate emotions measured in our survey, indicating that the set of specific climate emotions explored here are closely related (without redundancy) and lending support for the idea of a “web of emotions” as described by Jones and Davison (2021). This idea of a web of emotions is aligned with a move away from using single-item measures in climate emotions research (Stewart, 2021), for example, the Climate Anxiety Scale (Wullenkord et al., 2021). We echo these calls and see value in the CES used here, which included 11 items, as a reliable tool that could be used in other research and other settings to enhance understanding of climate emotions generally and to explore the relationship between climate emotions and endpoints like mental health or support for climate action. Future research testing and validating the CES used here is necessary to continue advancing research in this realm.

The results by gender offer an interesting insight into differences in how men and women feel about and respond to climate change. Using the CES, we found that women in the context of Canada’s Provincial North are experiencing climate emotions at higher levels than men. However, there were no significant differences across generations or parental status. du Bray et al. (2017) have also illustrated significant gendered patterns in terms of the emotional impacts of climate change across communities at heightened vulnerability to climate change. These findings emphasize the need for support and resources developed explicitly for/with women concerning coping with, managing, and processing climate emotions and emotional distress. Moreover, these findings indicate a potential heightened risk of mental health consequences among women as the climate crisis unfolds. Future research should aim to conduct more robust gender-based analyses and determine which kinds of support and resources women want and need to prevent impacts on overall mental health and well-being.

Although existing research has illustrated and argued that youth and young adults likely experience greater emotional responses and impacts due to the climate crisis (Hickman et al., 2021), climate anxiety, in particular, is not seen in our study setting. This divergence from existing scholarship on climate emotions could be due, in part, to the fact that our sample only included adults aged 18 and up; therefore, the experiences of youth and children are not captured in our data. This divergence could also be explained, in part, by the strong connectedness to land and nature that is common and characterizes the Provincial North (Galway et al., 2021), which likely enhances experiences of emotional distress in relation to climate change and increases with age and living in a particular community for a long period (which is the case for our sample). However, our results regarding age are in line with Wullenkord et al. (2021) who found no bivariate correlation between age and climate anxiety specifically. Additionally, although we expected to see a correlation between climate emotion and parental status, our results did not show this.

Informed by emerging literature and the findings reported here, we offer the concept of a *constellation of climate emotions.* This concept captures the diversity of emotions that people are experiencing in relation to climate change, intentionally brings to mind the interconnectedness among diverse emotions, and helps to move away from the positive/negative dichotomy concerning climate emotions that is common in existing research. Although this positive/negative binary is perhaps helpful in some ways for describing and theorizing about climate emotions broadly, it is important to note that this dichotomy and language may be limiting and even problematic. So-called “negative” emotions are rational and justified given the realities of the climate crisis and are often generative in that they can inspire conversation, engagement, and action related to climate change (Pihkala, 2018; Galway et al., 2019). Although emotions like worry, frustration, anger, sadness, and helplessness may indeed be challenging to bear and process, positioning these climate emotions as negative may encourage people and communities to turn away from, ignore, or silence these emotions impacts and the response of climate change rather than engage with and process them. At this time in human history, we are called upon to recognize and process our individual and collective climate emotions.

To conclude, this study has generated novel understanding of climate emotions in a specific regional context where no previous research existed. Findings highlight widespread and strong emotional responses in relation to climate change and indicate high levels of emotional distress overall. Two outcomes of this research deserve greater attention: first, the question of differential emotional response to climate by gender, and second, the idea of a constellation of climate emotions. The gender findings have potential implications for women’s health worldwide. If the climate crisis leads to high levels of emotional distress for women, this may interact with issues of gender inequity and health already negatively impacting women’s lives across the planet (World Health Organization [WHO], 2022). The second outcome of particular importance is the idea of a constellation of climate emotions. Considering a set of overlapping and potentially interacting emotional responses supports a more robust conception of emotional response to climate. Moreover, recognizing the adaptative nature of emotions allows for seeing certain emotions, such as anger, not as negative but as a rational and wholly justified response to this crisis of unprecedented proportion.

## Data Availability Statement

The original contributions presented in this study are included in the article/supplementary material, further inquiries can be directed to the corresponding author/s.

## Ethics Statement

The studies involving human participants were reviewed and approved by the Research Ethics Boards at Lakehead University, University of Northern British Columbia, and Simon Fraser University prior to data collection (code: 1466071; Date of approval: 2017/12/04). The patients/participants provided their written informed consent to participate in this study.

## Author Contributions

LG designed the “Climate Change Communication and Engagement in Canada’s Provincial North” project and led the survey instrument, postal survey implementation, and data collection. TB led data analysis and interpretation. LG and TB led the manuscript writing. Both authors read and agreed to the published version of the manuscript.

## Conflict of Interest

The authors declare that the research was conducted in the absence of any commercial or financial relationships that could be construed as a potential conflict of interest.

## Publisher’s Note

All claims expressed in this article are solely those of the authors and do not necessarily represent those of their affiliated organizations, or those of the publisher, the editors and the reviewers. Any product that may be evaluated in this article, or claim that may be made by its manufacturer, is not guaranteed or endorsed by the publisher.
